# 5-Chloro-2-hy­droxy­benzoic acid

**DOI:** 10.1107/S1600536810042042

**Published:** 2010-10-23

**Authors:** Abdul Rauf Raza, Bushra Nisar, M. Nawaz Tahir, Ahmad Raza

**Affiliations:** a Department of Chemistry, University of Sargodha, Sargodha, Pakistan; b Department of Physics, University of Sargodha, Sargodha, Pakistan

## Abstract

The asymmetric unit of the title compound, C_7_H_5_ClO_3_, contains two mol­ecules; both feature an intra­molecular O—H⋯O hydrogen bond, which generates an *S*(6) ring. In the crystal, both mol­ecules form inversion dimers linked by pairs of O—H⋯O hydrogen bonds with *R*_2_^2^(8) ring motifs. The dimers are inter­linked by C—H⋯O inter­actions.

## Related literature

For biological background, see: Bright *et al.* (2010 ▶): Fattorusso *et al.* (2005 ▶); Miki *et al.* (2002 ▶). For a related structure, see: Raza *et al.* (2010 ▶). For graph-set notation, see: Bernstein *et al.* (1995 ▶).
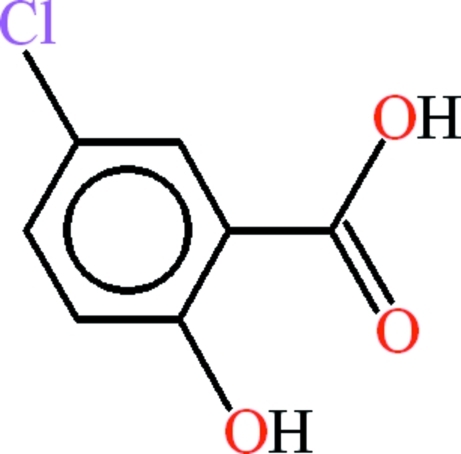


## Experimental

### 

#### Crystal data

C_7_H_5_ClO_3_
*M* *_r_* = 172.56
Monoclinic, 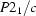

*a* = 23.526 (2) Å
*b* = 3.7972 (4) Å
*c* = 16.7321 (16) Å
β = 104.852 (5)°
*V* = 1444.8 (2) Å^3^
*Z* = 8
Mo *K*α radiation
μ = 0.48 mm^−1^
*T* = 296 K
0.34 × 0.12 × 0.10 mm

#### Data collection

Bruker Kappa APEXII CCD diffractometer
Absorption correction: multi-scan (*SADABS*; Bruker, 2005 ▶) *T*_min_ = 0.879, *T*_max_ = 0.888
14048 measured reflections
3697 independent reflections
2444 reflections with *I* > 2σ(*I*)
*R*_int_ = 0.047

#### Refinement

*R*[*F* ^2^ > 2σ(*F* ^2^)] = 0.042
*wR*(*F* ^2^) = 0.138
*S* = 1.03
3697 reflections
211 parameters
H atoms treated by a mixture of independent and constrained refinement
Δρ_max_ = 0.38 e Å^−3^
Δρ_min_ = −0.50 e Å^−3^

### 

Data collection: *APEX2* (Bruker, 2007 ▶); cell refinement: *SAINT* (Bruker, 2007 ▶); data reduction: *SAINT*; program(s) used to solve structure: *SHELXS97* (Sheldrick, 2008 ▶); program(s) used to refine structure: *SHELXL97* (Sheldrick, 2008 ▶); molecular graphics: *ORTEP-3* (Farrugia, 1997 ▶) and *PLATON* (Spek, 2009 ▶); software used to prepare material for publication: *WinGX* (Farrugia, 1999 ▶) and *PLATON*.

## Supplementary Material

Crystal structure: contains datablocks global, I. DOI: 10.1107/S1600536810042042/hb5691sup1.cif

Structure factors: contains datablocks I. DOI: 10.1107/S1600536810042042/hb5691Isup2.hkl

Additional supplementary materials:  crystallographic information; 3D view; checkCIF report

## Figures and Tables

**Table 1:** Hydrogen-bond geometry (Å, °)

*D*—H⋯*A*	*D*—H	H⋯*A*	*D*⋯*A*	*D*—H⋯*A*
O1—H1⋯O2^i^	0.83 (3)	1.88 (3)	2.710 (2)	171 (3)
O3—H3⋯O2	0.80 (3)	1.92 (3)	2.620 (2)	146 (3)
O4—H4*A*⋯O5^ii^	0.93 (3)	1.76 (3)	2.694 (2)	175 (2)
O6—H6⋯O5	0.87 (3)	1.80 (3)	2.606 (2)	154 (3)
C5—H5⋯O6^iii^	0.93	2.55	3.311 (3)	139

Symmetry codes: (i) 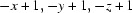; (ii) 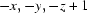; (iii) 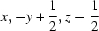.

**Fig. 1.**
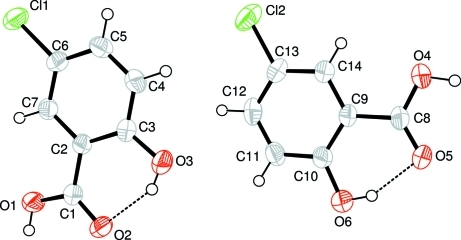
View of the title compound with displacement ellipsoids drawn at the 50% probability level. H-atoms are shown by small circles of arbitrary radius. The dotted lines indicate the intramolecular H-bonds.

**Fig. 2.**
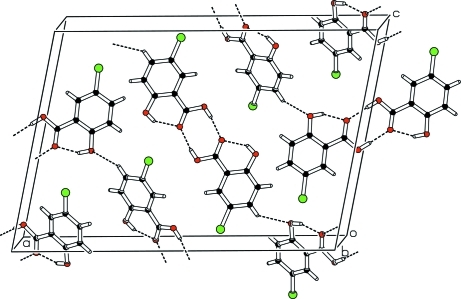
The partial packing (PLATON; Spek, 2009) which shows that molecules form dimers and are interlinked.

## supplementary crystallographic information

### Comment

The benzoxazepines have a plethora of biological activities ranging from anti-inflammatory effect (Miki *et al.*, 2002) to degenerative diseases like AIDS (Fattorusso *et al.*, 2005) and cancer (Bright *et al.*, 2010). Salicylic acid is an attractive substrate for the synthesis of 4,1-benzoxazepine. The objective of this work is to synthesize a variety of substituted salicylic acid derivatives as precursors for the asymmetric synthesis of 4,1-benzoxazepines by chiral-pool strategy.

We have reported the crystal structure of 2-methylamino-5-nitrobenzoic acid (Raza *et al.*, 2010) and in continuation to synthesize substituted benzoic acid, the title compound (I, Fig. 1) is being reported.

The title compound consists of two molecules in the crystallographic asymmetric unit which differ from each other geometrically. Both molecules, A (C1—C7/O1/O2/O3/CL1) and B (C8—C14/O4/O5/O6/CL2) are close to planar with r. m. s deviations of 0.023 and 0.007 Å, respectively. The dihedral angle between A/B is 1.77 (4)°. In each molecule, there exists an S(6) ring motif (Bernstein *et al.*, 1995) due to intramolecular H-bonding of O—H···O type (Table 1, Fig. 1). The molecules form dimers with themselves due to intermolecular H-bondings of O—H···O type (Table 1, Fig. 2) with *R*_2_^2^(8) ring motifs. These dimers are interlinked with each other due to H-bonding of C—H···O type (Fig. 2).

### Experimental

A solution of Cu_2_Cl_2_ (3.46 g, 0.0375 mol) in HCl (10 ml) was added as drops to the diazonium salt of 5-amino-2-hydroxybenzioc acid (3.825 g, 0.025 mol), which was prepared by adding ice chilled aqueous solution of NaNO_2_ (2.58 g, 0.0375 mol) to the solution of 5-amino-2-hydroxybenzoic acid in EtOAc and H_2_SO_4_ (2.8 ml, 4.9 g, 0.05 mol). The temperature of the reaction mixture was controlled below 268 K. After the complete addition of Cu_2_Cl_2_, the reaction mixture was refluxed for one hour, cooled to room temperature, neutralized with aqueous NaHCO_3_ (10%) and extracted with EtOAc (3 × 25 ml). The organic layer was combined, dried over anhydrous Na_2_SO_4_, filtered, concentrated under reduced pressure and left for 48 h to afford light yellow needles of (I).

### Refinement

The coordinates of hydroxy H-atoms are refined. The aryl H-atoms were positioned geometrically with (C—H = 0.93 Å) and refined as riding with *U*_iso_(H) = *xU*_eq_(C, O), where *x* = 1.2 for all H atoms.

### Crystal data

**Table:**

C_7_H_5_ClO_3_	*F*(000) = 704
*M**_r_* = 172.56	*D*_x_ = 1.587 Mg m^−^^3^
Monoclinic, *P*2_1_/*c*	Mo *K*α radiation, λ = 0.71073 Å
Hall symbol: -P 2ybc	Cell parameters from 931 reflections
*a* = 23.526 (2) Å	θ = 2.8–26.0°
*b* = 3.7972 (4) Å	µ = 0.48 mm^−^^1^
*c* = 16.7321 (16) Å	*T* = 296 K
β = 104.852 (5)°	Needle, light yellow
*V* = 1444.8 (2) Å^3^	0.34 × 0.12 × 0.10 mm
*Z* = 8	

### Data collection

**Table:**

Bruker Kappa APEXII CCD diffractometer	3697 independent reflections
Radiation source: fine-focus sealed tube	2444 reflections with *I* > 2σ(*I*)
graphite	*R*_int_ = 0.047
Detector resolution: 7.40 pixels mm^-1^	θ_max_ = 28.7°, θ_min_ = 3.6°
ω scans	*h* = −31→31
Absorption correction: multi-scan (*SADABS*; Bruker, 2005)	*k* = −4→5
*T*_min_ = 0.879, *T*_max_ = 0.888	*l* = −22→22
14048 measured reflections	

### Refinement

**Table:**

Refinement on *F*^2^	Primary atom site location: structure-invariant direct methods
Least-squares matrix: full	Secondary atom site location: difference Fourier map
*R*[*F*^2^ > 2σ(*F*^2^)] = 0.042	Hydrogen site location: inferred from neighbouring sites
*wR*(*F*^2^) = 0.138	H atoms treated by a mixture of independent and constrained refinement
*S* = 1.02	*w* = 1/[σ^2^(*F*_o_^2^) + (0.073*P*)^2^] where *P* = (*F*_o_^2^ + 2*F*_c_^2^)/3
3697 reflections	(Δ/σ)_max_ < 0.001
211 parameters	Δρ_max_ = 0.38 e Å^−^^3^
0 restraints	Δρ_min_ = −0.50 e Å^−^^3^

### Special details

**Table:**

Geometry. Bond distances, angles *etc*. have been calculated using the rounded fractional coordinates. All su's are estimated from the variances of the (full) variance-covariance matrix. The cell e.s.d.'s are taken into account in the estimation of distances, angles and torsion angles
Refinement. Refinement of *F*^2^ against ALL reflections. The weighted *R*-factor *wR* and goodness of fit *S* are based on *F*^2^, conventional *R*-factors *R* are based on *F*, with *F* set to zero for negative *F*^2^. The threshold expression of *F*^2^ > σ(*F*^2^) is used only for calculating *R*-factors(gt) *etc*. and is not relevant to the choice of reflections for refinement. *R*-factors based on *F*^2^ are statistically about twice as large as those based on *F*, and *R*- factors based on ALL data will be even larger.

### Fractional atomic coordinates and isotropic or equivalent isotropic displacement parameters (Å^2^)

**Table:**

	*x*	*y*	*z*	*U*_iso_*/*U*_eq_	
Cl1	0.37512 (3)	0.69966 (16)	0.07736 (3)	0.0501 (2)	
O1	0.49060 (7)	0.6286 (5)	0.38781 (10)	0.0531 (6)	
O2	0.43512 (7)	0.3536 (5)	0.45885 (9)	0.0497 (5)	
O3	0.32656 (7)	0.1864 (5)	0.38353 (10)	0.0504 (6)	
C1	0.44188 (9)	0.4711 (6)	0.39317 (12)	0.0372 (6)	
C2	0.39581 (8)	0.4495 (5)	0.31555 (11)	0.0325 (6)	
C3	0.34043 (9)	0.3106 (5)	0.31526 (13)	0.0363 (6)	
C4	0.29691 (10)	0.3000 (6)	0.24180 (14)	0.0427 (7)	
C5	0.30768 (9)	0.4181 (6)	0.16973 (13)	0.0425 (7)	
C6	0.36245 (9)	0.5504 (5)	0.16965 (12)	0.0359 (6)	
C7	0.40631 (9)	0.5677 (5)	0.24125 (12)	0.0355 (6)	
Cl2	0.13798 (3)	0.73267 (17)	0.21674 (4)	0.0624 (3)	
O4	0.01191 (7)	0.1719 (5)	0.39980 (11)	0.0604 (6)	
O5	0.06701 (7)	0.1273 (5)	0.52956 (10)	0.0553 (6)	
O6	0.17545 (7)	0.3485 (5)	0.56545 (10)	0.0538 (6)	
C8	0.06128 (9)	0.2160 (6)	0.45705 (14)	0.0402 (7)	
C9	0.10935 (9)	0.3749 (5)	0.42825 (12)	0.0349 (6)	
C10	0.16403 (9)	0.4322 (6)	0.48436 (13)	0.0383 (7)	
C11	0.20911 (9)	0.5788 (6)	0.45616 (14)	0.0447 (7)	
C12	0.20146 (10)	0.6683 (6)	0.37478 (15)	0.0453 (8)	
C13	0.14736 (10)	0.6137 (6)	0.31970 (13)	0.0413 (7)	
C14	0.10148 (9)	0.4679 (6)	0.34525 (13)	0.0397 (7)	
H1	0.5163 (12)	0.635 (7)	0.4326 (18)	0.0636*	
H3	0.3557 (12)	0.189 (7)	0.4210 (18)	0.0604*	
H4	0.25996	0.21164	0.24137	0.0512*	
H5	0.27810	0.40924	0.12074	0.0510*	
H7	0.44297	0.65761	0.24065	0.0425*	
H4A	−0.0156 (12)	0.058 (8)	0.4222 (16)	0.0725*	
H6	0.1424 (13)	0.258 (7)	0.5696 (19)	0.0646*	
H11	0.24542	0.61752	0.49328	0.0536*	
H12	0.23233	0.76478	0.35677	0.0543*	
H14	0.06536	0.43138	0.30750	0.0476*	

### Atomic displacement parameters (Å^2^)

**Table:**

	*U*^11^	*U*^22^	*U*^33^	*U*^12^	*U*^13^	*U*^23^
Cl1	0.0588 (4)	0.0597 (4)	0.0332 (3)	−0.0012 (3)	0.0142 (3)	0.0039 (2)
O1	0.0349 (9)	0.0858 (13)	0.0354 (9)	−0.0154 (8)	0.0034 (7)	0.0057 (8)
O2	0.0443 (9)	0.0749 (11)	0.0300 (8)	−0.0096 (8)	0.0096 (7)	0.0028 (7)
O3	0.0427 (10)	0.0719 (11)	0.0399 (9)	−0.0131 (8)	0.0167 (7)	0.0048 (8)
C1	0.0340 (11)	0.0433 (12)	0.0359 (10)	−0.0004 (9)	0.0119 (9)	−0.0002 (9)
C2	0.0314 (10)	0.0349 (10)	0.0318 (10)	0.0006 (8)	0.0092 (8)	−0.0018 (8)
C3	0.0356 (11)	0.0370 (11)	0.0395 (11)	−0.0004 (9)	0.0153 (9)	−0.0010 (8)
C4	0.0324 (11)	0.0477 (13)	0.0473 (13)	−0.0058 (9)	0.0091 (10)	−0.0004 (9)
C5	0.0358 (12)	0.0474 (13)	0.0405 (12)	−0.0017 (10)	0.0028 (9)	−0.0011 (9)
C6	0.0408 (11)	0.0353 (10)	0.0319 (10)	0.0017 (9)	0.0098 (9)	−0.0008 (8)
C7	0.0331 (11)	0.0386 (11)	0.0358 (10)	−0.0009 (9)	0.0109 (8)	−0.0028 (8)
Cl2	0.0825 (5)	0.0671 (5)	0.0442 (3)	−0.0108 (3)	0.0284 (3)	0.0069 (3)
O4	0.0345 (9)	0.0962 (14)	0.0511 (10)	−0.0143 (9)	0.0121 (8)	0.0148 (9)
O5	0.0414 (9)	0.0852 (13)	0.0429 (9)	−0.0100 (8)	0.0173 (7)	0.0136 (8)
O6	0.0402 (9)	0.0821 (12)	0.0387 (9)	−0.0076 (9)	0.0093 (7)	0.0030 (8)
C8	0.0313 (11)	0.0481 (13)	0.0447 (12)	0.0001 (9)	0.0159 (10)	0.0023 (9)
C9	0.0326 (11)	0.0374 (11)	0.0386 (11)	0.0008 (9)	0.0163 (9)	0.0002 (8)
C10	0.0357 (11)	0.0429 (12)	0.0378 (11)	0.0017 (9)	0.0123 (9)	−0.0023 (9)
C11	0.0317 (11)	0.0528 (14)	0.0499 (13)	−0.0065 (10)	0.0113 (10)	−0.0034 (10)
C12	0.0413 (13)	0.0420 (12)	0.0597 (15)	−0.0090 (10)	0.0261 (11)	−0.0056 (10)
C13	0.0517 (14)	0.0385 (11)	0.0400 (11)	−0.0019 (10)	0.0230 (10)	−0.0004 (9)
C14	0.0374 (11)	0.0450 (12)	0.0386 (11)	−0.0018 (9)	0.0130 (9)	0.0005 (9)

### Geometric parameters (Å, °)

**Table:**

Cl1—C6	1.741 (2)	C4—C5	1.370 (3)
Cl2—C13	1.740 (2)	C5—C6	1.383 (3)
O1—C1	1.316 (3)	C6—C7	1.368 (3)
O2—C1	1.234 (3)	C4—H4	0.9300
O3—C3	1.351 (3)	C5—H5	0.9300
O1—H1	0.83 (3)	C7—H7	0.9300
O3—H3	0.80 (3)	C8—C9	1.468 (3)
O4—C8	1.313 (3)	C9—C14	1.399 (3)
O5—C8	1.233 (3)	C9—C10	1.402 (3)
O6—C10	1.352 (3)	C10—C11	1.383 (3)
O4—H4A	0.93 (3)	C11—C12	1.370 (3)
O6—H6	0.87 (3)	C12—C13	1.382 (3)
C1—C2	1.465 (3)	C13—C14	1.375 (3)
C2—C7	1.402 (3)	C11—H11	0.9300
C2—C3	1.404 (3)	C12—H12	0.9300
C3—C4	1.384 (3)	C14—H14	0.9300
			
C1—O1—H1	113.3 (19)	C6—C7—H7	120.00
C3—O3—H3	108 (2)	C2—C7—H7	120.00
C8—O4—H4A	109.9 (16)	O5—C8—C9	122.4 (2)
C10—O6—H6	103 (2)	O4—C8—C9	115.14 (19)
O1—C1—C2	115.11 (17)	O4—C8—O5	122.4 (2)
O2—C1—C2	122.3 (2)	C8—C9—C14	120.85 (19)
O1—C1—O2	122.58 (19)	C8—C9—C10	119.74 (18)
C1—C2—C7	120.64 (18)	C10—C9—C14	119.4 (2)
C3—C2—C7	119.43 (18)	O6—C10—C11	117.7 (2)
C1—C2—C3	119.93 (17)	O6—C10—C9	123.2 (2)
C2—C3—C4	119.24 (19)	C9—C10—C11	119.09 (19)
O3—C3—C4	117.2 (2)	C10—C11—C12	121.5 (2)
O3—C3—C2	123.58 (19)	C11—C12—C13	119.3 (2)
C3—C4—C5	120.7 (2)	Cl2—C13—C12	118.86 (18)
C4—C5—C6	120.2 (2)	Cl2—C13—C14	120.14 (17)
Cl1—C6—C7	119.94 (17)	C12—C13—C14	121.0 (2)
C5—C6—C7	120.69 (19)	C9—C14—C13	119.7 (2)
Cl1—C6—C5	119.37 (16)	C10—C11—H11	119.00
C2—C7—C6	119.77 (19)	C12—C11—H11	119.00
C5—C4—H4	120.00	C11—C12—H12	120.00
C3—C4—H4	120.00	C13—C12—H12	120.00
C4—C5—H5	120.00	C9—C14—H14	120.00
C6—C5—H5	120.00	C13—C14—H14	120.00
			
O1—C1—C2—C3	−175.24 (19)	O4—C8—C9—C10	−179.8 (2)
O1—C1—C2—C7	4.5 (3)	O4—C8—C9—C14	−0.2 (3)
O2—C1—C2—C3	4.0 (3)	O5—C8—C9—C10	−0.4 (3)
O2—C1—C2—C7	−176.3 (2)	O5—C8—C9—C14	179.1 (2)
C1—C2—C3—O3	−1.1 (3)	C8—C9—C10—O6	−0.1 (3)
C1—C2—C3—C4	178.4 (2)	C8—C9—C10—C11	179.3 (2)
C7—C2—C3—O3	179.12 (19)	C14—C9—C10—O6	−179.7 (2)
C7—C2—C3—C4	−1.3 (3)	C14—C9—C10—C11	−0.2 (3)
C1—C2—C7—C6	−178.98 (19)	C8—C9—C14—C13	−179.5 (2)
C3—C2—C7—C6	0.8 (3)	C10—C9—C14—C13	0.1 (3)
O3—C3—C4—C5	−179.4 (2)	O6—C10—C11—C12	179.4 (2)
C2—C3—C4—C5	1.0 (3)	C9—C10—C11—C12	−0.1 (3)
C3—C4—C5—C6	−0.1 (3)	C10—C11—C12—C13	0.5 (3)
C4—C5—C6—Cl1	−179.91 (18)	C11—C12—C13—Cl2	179.34 (18)
C4—C5—C6—C7	−0.5 (3)	C11—C12—C13—C14	−0.7 (3)
Cl1—C6—C7—C2	179.56 (15)	Cl2—C13—C14—C9	−179.65 (17)
C5—C6—C7—C2	0.2 (3)	C12—C13—C14—C9	0.4 (3)

### Hydrogen-bond geometry (Å, °)

**Table:**

*D*—H···*A*	*D*—H	H···*A*	*D*···*A*	*D*—H···*A*
O1—H1···O2^i^	0.83 (3)	1.88 (3)	2.710 (2)	171 (3)
O3—H3···O2	0.80 (3)	1.92 (3)	2.620 (2)	146 (3)
O4—H4A···O5^ii^	0.93 (3)	1.76 (3)	2.694 (2)	175 (2)
O6—H6···O5	0.87 (3)	1.80 (3)	2.606 (2)	154 (3)
C5—H5···O6^iii^	0.93	2.55	3.311 (3)	139

Symmetry codes: (i) −*x*+1, −*y*+1, −*z*+1; (ii) −*x*, −*y*, −*z*+1; (iii) *x*, −*y*+1/2, *z*−1/2.
